# On the Language of Complaint during Pain, and the Consequences of Suppressing It, Principally from the French of Baron Larrey

**Published:** 1819-07-01

**Authors:** 


### *On the Language of Complaint during Pain, and the Consequences of suppressing it, principally from the French of* Baron Larrey.

THE life of man, from the instant of his birth till the moment of his dissolution, is one continued struggle against the innumerable dolorific agents that surround and annoy him. The same organs of sense by which he enjoys pleasure, are the media through which pain is also conveyed. That the sense of pain is as wisely implanted in our nerves as the sense of pleasure, there can be little doubt; but our present object is to inquire whether that peculiar language of complaint (which, indeed, is the universal language of all animated nature) has any *salutary* tendency, or is merely the index of our sensations.

The infant cries the moment it enters this world; no doubt from a sense of pain occasioned by the new impression of the atmosphere. It has no other language to make known its pains or uneasiness; but in this view alone, it is a wise gift of the Omnipotent Creator. Were it not for this animated language of Nature, the careless nurse might implant pins into the infant's flesh, or neglect to supply it with sufficient aliment. As nature seldom adapts a single mean to a single purpose, we have every reason to believe that the *moderate action of crying* has a salutary influence on the pulmonary system of the child, and through that on the whole corporeal fabric.

That the language of complaint is useful in *moral* afflictions, we have the most demonstrative evidence. The fact is so striking as to have arrested the attention of the poets as well as the physicians of every age. A flood of *tears* instantly relieves both mind and body! The cheeks of Niobe were dry when grief burst her heart for the loss of her children. The observant physician sees, at at every step of his professional rounds, the morbid corporeal effects of *suppressed* mental emotions. This did not escape the penetrating eye of Shakespeare:

> ———————— "She never told her love,
> But let Concealment, like a worm i' the bud,
> Feed on her damask cheek."

It is in this way that the confidence of a friend, to whom we can unbosom our sorrows, often prevents that *corporeal* derangement in the brain, which ends in insanity or suicide, when the cause of grief is kept locked up in the breast. It is a certain fact, that even *solitude* confers a comparative relief, because we are there, as it were, enabled to give vent to our emotions in sighs, groans, or ejaculations. But when in the midst of company, to whom we dare not communicate our thoughts, we endure the pangs of the damned, and are always eager to get away into the open air by ourselves.

The ravages which grief and other melancholy emotions make upon the function and structure of our corporeal organs, is truly astonishing. They are not much less numerous than those from *physical* causes And why need we wonder at this, when we have so many well authenticated instances of death itself, on the spot, from mental emotions? Marcellus Donatus relates, that at the Siege of Buda, a young soldier fought with such surprising valour, as to excite the admiration of the whole army, both friends and enemies; but who at length fell, overpowered by numbers. It was eagerly inquired who this valiant youth could be; and after the battle, his body being found, and his vizor taken off, Raisciat of Suabia recognized him as his son! The father remained perfectly motionless for a few seconds, his eyes tearless and fixed on the corpse—he then fell over the body, and instantly expired without uttering a groan!

Innumerable instances of the effects of sorrow, though less suddenly fatal, on the functions and structure of the viscera might be here adduced; but we shall only state one or two. Boyle relates that a woman having sat down to rest on the bank of a canal, permitted her infant child to creep about on the grass. The child however fell into the water, and the mother on looking up saw her offspring sinking! She was instantly stricken with palsy of one arm, which continued during life.

But what intelligent or experienced physician has not seen hypochondriasis, mania, epilepsy, result from moral afflictions? It is, however, on the *epigastric centre* that grief and anxiety chiefly vend their morbid effects. Hence it is that aneurism and other structural derangements of the heart are so frequently developed; and hence too those scirrhous affections of the stomach, especially of the pyloris, which we meet with among people long preyed on by melancholy emotions of the mind.

The hepatic system also comes very particularly under the influence of suppressed sorrow, and strong mental conflicts. We have seen an incredible number of these cases. Renauldin relates the case of a man who, in one minute was suffused completely yellow, from mental agitation, under his own eye. But to return to the language of complaint in painful diseases, and during operations.

The proud stoic will tell you, that as pain is not an evil, to complain is unworthy of a man; and to *cry* is a piece of shameful pusillanimity. But the stoic, instead of being a natural, is a most *unnatural* philosopher! Pain is the lot of human nature; but the language of complaint is permitted to him who suffers. Pain, indeed, very generally tames the proudest and the fiercest mind; like the force of love, it reduces all ranks to a level. When Felix operated on Henry the 4th, of France, for a *fistula in ano*, the patient at first endeavoured to suppress his feelings, and act the monarch. But the bistoury soon subdued his resolution, and before the operation was over, this prince and hero roared as loud as any of his subjects!

To *moderate* the expression of our feelings in painful diseases, and especially under surgical operations, is commendable; to *suppress* them is dangerous. It is not beneath the dignity of the bravest warrior to wince under the surgeon's knife. Read the Iliad, and your ears will be stunned with the cries of the Grecian and Trojan heroes when wounded. In fact, the *experienced* surgeon will be far from gratified to find his patient utter not a complaint during a painful surgical operation. It is a bad omen, whether it result from insensibility of the patient, or a proud suppression of his feelings. Anne, of Austria, was afflicted with a cancer in the breast, which caused the most excruciating torture at intervals. Her confessor exhorted her to restrain her expressions of sufferings at these times; but in doing so, she experienced such a sense of suffocation and anguish internally, as nearly overwhelmed her. The Marechal du Muy was operated on for stone in the bladder. He prayed devoutly at mass, just before the operation, for strength of mind to bear the pain; and he so far suppressed the feelings of nature, that he uttered not a murmur while the stone was extracted. He died almost immediately afterwards.

Baron Percy, who has performed as many surgical operations as any man living, remarks, that "it is a favourable circumstance when, under great operations, the patient cries and sheds tears. It appears that, in such cases, a kind of general relaxation takes place in the system, rendered previously almost convulsed by the pain. The functions of the heart are deranged; the respiration is impeded; the blood is detained in the large vessels; the nervous system is spasmed; and the motions of the diaphragm are irregular and tumultuous. All these morbid phenomena are greatly exasperated, *if the language of complaint be suppressed;* [si le malade se tait et resiste.] The impression of anguish cannot be effaced afterwards; the vital powers take a destructive direction; the whole animal economy is perverted; and sudden death, or a low fever, too often ending in the same, is the sad result of this state of things." "L'empreinte de la douleur ne peut plus s'effacer; les propriétés vitales recoivent une direction destructive; L'organisme entier est etrainé dans cette perversion, et une mort prompte, ou une fievre ataxique dont elle est trop souvent la terminaison, est le triste résultat de cet état de chose." "If, on the contrary, the patient cries out; if he exhales; if, as Montagne says, he evaporates the pain, these effects will not take place, and these impressions will be but transitory. "Si le malade crie, s'il exhale, si, comme dit encore Montaigne, il evapore sa douleur, ces effets sont presque nuls, et ces impressions simplement fugitives." Every cry, which consists in a deep inspiration followed by a sudden and interrupted expiration, dilates and distends every part which the pain had previously constricted; thus preventing congestions; facilitating the circulation of the blood through the lungs; disembarrassing the heart: in fine, bringing constantly back that *order*, which pain, the great enemy of order, tends incessantly to subvert.

The Baron remarks, that he has often met with men who, in the midst of the most terrible sufferings, knew not how to, or could not cry out. They appeared as if stupified, or absorbed by the pain, which seemed to reign paramount over every other sentiment and instinct. " I have, says this experienced surgeon exhorted men, under these circumstances, to cry aloud, when I saw the pain ravaging on their vitals; their necks and chests swell; the hair bristle on their heads; the mouth closed, and distorted; all the muscles contracted; the face pale, and sunk; the eye fixed and projected—the countenance wild; a hollow, stertorous sound issuing from the bottom of the throat; and yet suffering nature giving no vent to her feelings."—This may be termed the *passive* suppression of complaint; for when from false pride, or other motive, men have constrained by violence their feelings, the phenomena wear a different aspect. The face becomes swelled and reddened—the abdomen is flattened and contracted—the hypochondria project—the mouth is open, as if to give passage to cries which are stifled in the throat—the eyes are red, injected, but not moistened with tears.—The whole body bears the marks of violent efforts to overcome the pain. But even these expressions of the countenance can sometimes be suppressed.

"I extirpated, (says Baron Larrey) the breast of a lady affected with cancer, who was of a most religious turn of mind. She held a crucifix in her hand, smiling and talking to it in the most tranquil and placid manner, during the whole of the operation, while her body was contorted with agony. She was seized immediately afterwards with a universal spasmodic affection, which nearly put an end to her existence. "Another time, says the same distinguished surgeon, I operated, for a large popliteal aneurism, on the master of a seminary who, though young, and apparently susceptible, bore the operation, which was very painful and complicated, without uttering a word, or moving a single muscle. But a terrible *re-action* succeeded this internal struggle between Nature and pain! Spasms and cramps assailed the suffering patient for the first fifteen days after the operation, and he did not recover under three months."

We trust that the observations contained in the foregoing paper, may not be unprofitable to the surgical reader. Every thing which can mar in any degree the success of an operation is of great importance to the young surgeon; and we have repeatedly seen much mischief done by the almost universal habit which surgeons are in of repressing the language of complaint in their patients, and exhorting them to bear their sufferings without a murmur. This evil is increased by the *bravado* of the patient, who, in an operation room or public hospital, endeavours to stifle his groans, and act the hero, when it would be much wiser to shew himself the frail mortal, the child of pain, by giving a moderate and natural vent to the LANGUAGE OF COMPLAINT.

---

